# Effect of microtitanium impregnated tape on the recovery of triceps surae musculotendinous function following strenuous running

**DOI:** 10.1186/2193-1801-2-653

**Published:** 2013-12-05

**Authors:** Jonathan D Hughes, Philip W Fink, David F Graham, David S Rowlands

**Affiliations:** Exercise and Sport Research Centre, University of Gloucestershire, Gloucester, UK; Rehabilitation Sciences, Griffith University, Gold Coast, Queensland Australia; School of Sport and Exercise, Massey University, Palmerston North, New Zealand; School of Sport and Exercise, Massey University Wellington, Wellington, New Zealand

**Keywords:** Tendon compliance, Recovery, Stretch reflex, Tendon tap, Dynamometry

## Abstract

We previously reported increased running economy and joint range of motion (ROM) during subsequent exercise performed 48-h following strenuous exercise while wearing garments containing micro-titanium particles generated from high-pressure aqueous processing of titanium (AQUA TITAN^TM^). Here we utilised an isolated plantarflexion triceps surae model and AQUA TITAN-treated flexible tape to determine if dermal application of the micro-titanium could account for meaningful changes in functional properties of the musculotendinous unit. In a randomised double-blind crossover, 20 trained men day 1, baseline measures, AQUA TITAN or placebo tape covering the triceps surae, intermittent high-intensity treadmill running; day 2, rest; day 3, post-stress post-treatment outcome measures. Outcomes comprised: plantarflexion ROM via isokinetic dynamometry; short latency reflex from electromyography; Achilles tendon stiffness from isometric dynamometry, ultrasonography (Achilles-medial-gastrocnemius junction), motion analysis, and force-length modelling. High-intensity exercise with placebo tape reduced tendon stiffness (-16.5%; 95% confidence limits ±8.1%; small effect size), relative to non-taped baseline, but this effect was negligible (-5.9%; ±9.2%) with AQUA TITAN (AQUA TITAN-placebo difference -11.3%; ±11.6%). Change in latency relative to baseline was trivial with placebo (1.6%; ±3.8%) but large with AQUA TITAN (-11.3%; ±3.3%). The effects on ROM with AQUA TITAN (1.6%; ±2.0%) and placebo were trivial (-1.6% ±1.9%), but the small difference (3.1%; ±2.7%) possibly greater with AQUA TITAN. AQUA TITAN tape accelerated the reflex response and attenuated reduced Achilles tendon stiffness following fatiguing exercise. Altered neuromuscular control of tendon stiffness via dermal application of micro-titanium treated materials may facilitate restoration of musculotendinous contractile performance following prior strenuous exercise.

## Introduction

There is considerable current interest in research, athletic, and rehabilitation fields for interventions that can improve musculotendinous function following fatiguing exercise. Recently, garments treated with titanium microparticles (AQUA TITAN™) worn during recovery from simulated soccer match play or hill running increased subsequent joint range of motion (ROM) (Wadsworth et al. 2010) and running economy (Rowlands et al. 2013), respectively, but the neural, musculotendinous, or other physiological mechanisms for these changes were not identified. Increased ROM and lower metabolic cost during subsequent activity could be a result of improved musculotendinous function attributable to changes in tendon compliance and/or dynamic neuromuscular performance reflected in the short latency reflex, which contributes to improved contractile performance during running (Ishikawa and Komi 2007).

Tendon stiffness plays an important role in many facets of movement. High tendon compliance can enhance the storage and release of energy during muscular contraction (Alexander 2002), while high stiffness maximizes force transfer (Lichtwark et al. 2007). During running, maximal muscle forces produced in the triceps surae are higher for athletes with stiffer tendons (Hof et al. 2002). In addition, economical runners possess higher contractile strength and tendon stiffness indicating that muscle-tendon unit functionality during running is dependent both on the stiffness of the series elastic component and on the maximal strength of the contractile component (Arampatzis et al. 2006). During recovery from a bout of exhaustive exercise, limb fatigue reduced subsequent running economy (Hunter and Smith 2007) which may be due to reduced muscle strength and tendon stiffness (Lichtwark et al. 2007) as occurring in high-load isometric models (Kay and Blazevich 2009).

In addition to potential changes in tendon function, AQUA TITAN may also impact on peripheral neuromuscular performance. In isolated mice hippocampal neurons, AQUA TITAN tape reduced the resting membrane potential and action potential frequency (Korte 2008). Since the rate of force development is an important determinant of muscle-tendon performance (Fukashiro et al. 1995), altered motor neuron firing rates with AQUA TITAN could improve unit contractile performance.

To more clearly define and quantify the magnitude of the effect of AQUA TITAN on musculotendinous function, a functionally relevant isolated triceps surae model of plantarflexion was used. Achilles tendon stiffness and reflex response were studied using dynamometry, motion analysis, ultrasonography and electromyography before and 48-h following a strenuous treadmill run (Drust et al. 2000). We hypothesised that AQUA TITAN tape would better maintain normal rested baseline muscle-tendon function in accordance with the *trained optima* concept of Lichtwark and Wilson (2007), as represented by attenuation of reflex function, strength, and stiffness during recovery from hard running, relative to placebo.

## Methods

Twenty trained male team sport athletes competing in regional level soccer, rugby, or field hockey competition, aged 26.6 ± 7.8 y (mean ± SD), and with mean body mass of 81.2 ± 11.3 kg, stature of 179 ± 4.4 cm, and maximum oxygen uptake of 61.6 ± 7.1 mL·kg^-1^·min^-1^, volunteered to participate in the study. A sample size of 20 was estimated for the expected effect size on Achilles tendon stiffness based on effect size estimate drawn from change in plantarflexion ROM due to AQUA TITAN of 3.7% (effect size 0.2) (Wadsworth et al. 2010) and the coefficient of variation for tendon length using similar procedures (6.1%) (Fletcher et al. 2010), and the method of sample size for meaningful (effect size) magnitude-based inference (Hopkins et al. 2009). Potential participants were interviewed and subsequently excluded if they had a history of Achilles tendinopathy or lower limb trauma, illness or were currently on analgesic medication. All participants were informed in writing about the potential risks of the study and gave written informed consent for their participation in the study, which was performed according to the Declaration of Helsinki and approved by the University’s Research Ethics Committee prior to the start of the investigation.

### Procedure

**Figure 1:**
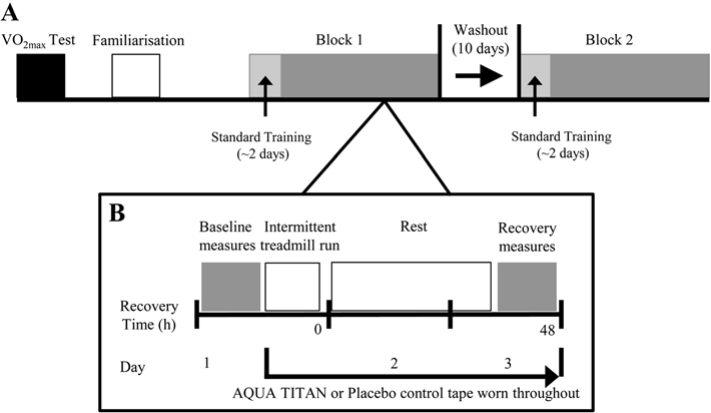
**Experimental design.** Shown is **(A)** pre-testing, familiarisation measures, and the two crossover blocks, followed by (**B**, inset) detail of one of the two 3-d experimental blocks.

All participants first completed a treadmill-based assessment of  O_2max_ (Wadsworth et al. 2010) followed 1-wk later by familiarization of the experimental procedures (Figure 1). Participants performed a double-blind randomised crossover comprising baseline measurements of joint range of motion (ROM), Achilles tendon stiffness, short latency reflex, and isokinetic torque, followed by a 40-min treadmill protocol to simulate the physical demands of intermittent high-intensity sport shown previously to cause leg muscle fatigue and altered contractility (Drust et al. 2000; Rahnama et al. 2006). The treadmill speeds for each activity in the protocol were based upon the speeds observed for each specific movement category during soccer match-play: walking 6 km·h^-1^, jogging 12 km·h^-1^, cruising km·h^-1^ and sprinting 21 km·h^-1^. The protocol was arranged around two identical cycles separated by a static recovery period of 90 s. Each cycle consisted of 23 discrete bouts of activity (duration): 6 × walking (35.3 s); 6 × jogging (50.3 s); 3 × cruising (51.4 s), and 8 × sprinting (10.5 s). High-intensity exercise (cruise and sprint) bouts were separated by low-intensity recovery (walk and jog), ordered via within-subject randomisation, and replicated in the second arm of the crossover. Recovery mechanics were assessed 48-h following the run after an intervening rest day. There was a 10-d washout between experimental blocks.

**Figure 2:**
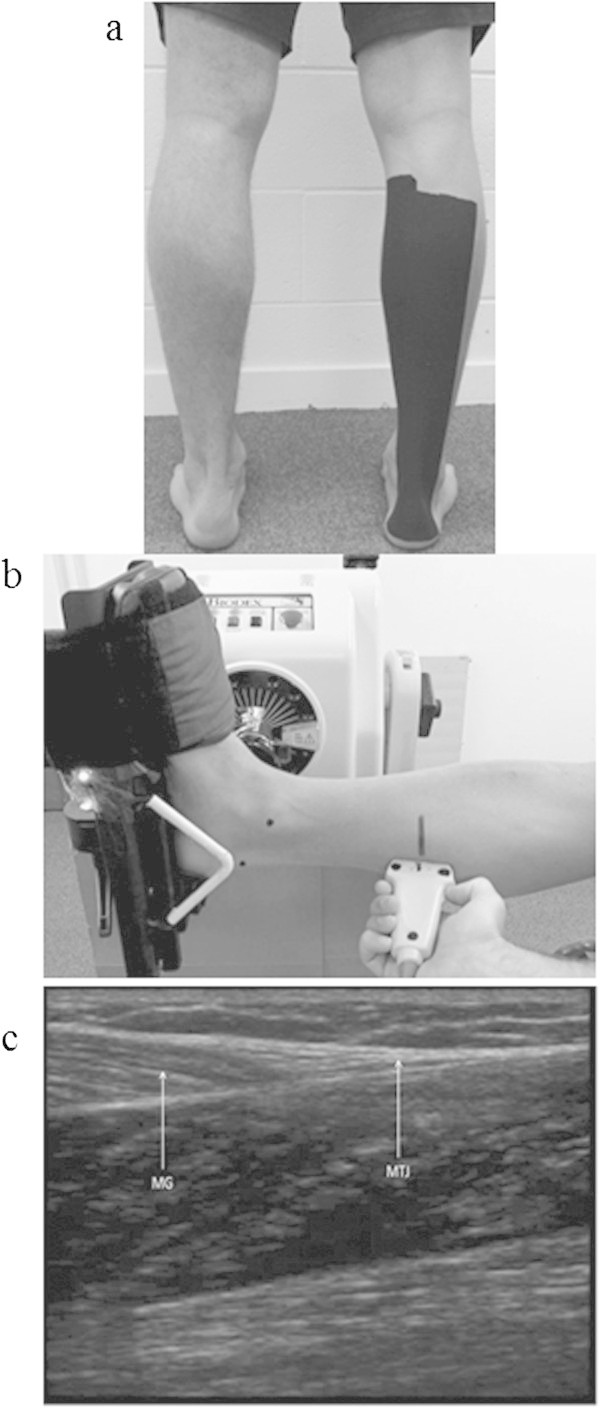
**Experimental set-up to determine the effect of AQUA TITAN tape on triceps surae contractile function.** Shown is **(a)** positioning of the experimental tape covering the entire region of the posterior lower limb (triceps surae), **(b)** the contrasting marker (motion analysis) and ultrasound probe positioning and LED synchronization marker, and **(c)** the ultrasound image of medial gastrocnemius (MG)–Achilles junction (MTJ).

All 20 participants completed one trial wearing AQUA TITAN treated tape and one wearing a placebo tape allocated by double-blind randomization. Both the AQUA TITAN and placebo tapes were custom made by Phiten Co. Ltd. (Kyoto, Japan), using an AQUA TITAN concentration of 540 ml·l^-1^ in the treatment tape. AQUA TITAN is a suspension of titanium nanoparticles in water (Hirata et al. 2004 Patent 522431). Phiten Co. Ltd also funded the study but had no other involvement in, or right to approve or disapprove the current publication. Both tapes were black and covered the entire posterior lower limb from the calcaneus to the proximal attachments of the medial and lateral gastrocnemius (Figure 2a). The tape was applied following the baseline measures after which it remained in place for the duration of the intermittent treadmill protocol and the entire recovery period including all exercise tests and while sleeping. The blinding code was maintained by an external party and revealed to researchers only after the analysis was performed. To prevent any possible mixing, tapes were stored separately.

The effect of the tape was assessed through dynamometry and analysis of the short latency reflex, in that order. Dynamometry was taken with participants reclined in a supine position at a hip angle of 0° (i.e. fully extended). The foot of the dominant leg was placed against the footplate of the dynamometer. The lever arm of the dynamometer was aligned so that the center of rotation of the dynamometer was aligned with the lateral malleolus. The initial ankle position was preset at neutral, 0°, mid plantar/dorsiflexion. Maximal plantar flexion isometric torque was sampled at six predefined angles (0, 5, 10, 15, 20 and 25°). Two contractions were performed with 120 s recovery between efforts. Participants were instructed to impart force to the foot plate in a gradual manner until they reach maximal effort around 1 s into the contraction then to hold that force for the remaining 2 s. Maximal plantar flexion isokinetic torque was sampled at four predefined angular velocities (30, 60, 90 and 120°·s^-1^) through a predetermined range of motion (25°). Three repetitions at each angular velocity were performed; each velocity was interspersed with 180 s recovery. The isometric conditions were used to estimate tendon stiffness, while the isokinetic data was used to measure the functional changes in the muscle/tendon complex.

Following the dynamometry, changes in the short latency reflex of the medial gastrocnemius muscle were assessed using a tendon tap method. Participants lay prone with the ankle at 90º passive dorsiflexion and were instructed to relax. The Achilles tendon was tapped with an instrumented reflex hammer operated by hand. This test was performed three times.

### Apparatus

An isokinetic dynamometer (Biodex Medical Systems System 3, NY, USA) was used to sample isometric tension and isokinetic torque. Participants were videoed while performing the dynamometer test using a Casio Exlim Ex-F1 camera (Casio Computer Co Ltd, Tokyo Japan) at 30 Hz. Black circles were placed on the Achilles insertion at the calcaneus, the medial malleolus and on the head of the ultrasound transducer (Figure 2b). In order to assess tendon length, ultrasound images of the medial gastrocnemius/Achilles tendon musculotendonous junction (MG/AT MTJ) were simultaneously collected at 10 Hz using a Sonosite, MicroMaxx ultrasound (Sonosite Inc., Bothell, USA).

Torque and position for the dynamometer were collected using an ADI power lab system (PowerLab 4/25, ADInstruments) at 1000 Hz. The ultrasound data collection was manually triggered; the trigger also activated a light emitting diode which was used as a synchronization event in the video image, and a signal was simultaneously sent to the power lab system to start data collection. The video was analyzed to find the first frame in which the light was visible, and this frame coincided with the first frame of the ultrasound image, as well as the first sample from the power lab. Using this method, the video was synchronized to within 1/30 of a second of the ultrasound and dynamometer.

The locations of the markers in the video and the MTJ in the ultrasound image were digitized using MaxTRAQ software (version 2.19-012, Innovision systems Inc. Columbiaville, MI, USA). The ultrasound probe was orientated along the longitudinal axis of the MG/AT MTJ. When the location of the MTJ was ascertained, the probe was positioned so that both the superficial and, importantly, the deep aponeurosis between MG and Soleus were apparent, ensuring accurate and reliable identification of the MTJ. To match the sampling frequency of the ultrasound, only every third frame of the video was digitized. To calculate the location of MG/AT MTJ in absolute space, the coordinates of the MTJ in the ultrasound image were added to the centre point of the ultrasound probe digitized in the video.

To examine the short latency reflex, surface electromyographic (sEMG) activities of the GM muscles were recorded from the right leg using bipolar surface electrodes with a 5 mm diameter and a 10 mm fixed inter-electrode distance (Ambu® Blue Sensor N, Ambu A/S, Ballerup Denmark). Skin preparation and electrode placement were performed according to international guidelines for sensor placement (Hermens et al. 2000). sEMG signals were sampled at 1000 Hz during tendon tap. The Achilles tendon was tapped with an instrumented hammer (ADInstruments, Australia). The sEMG and hammer signal were collected using an ADI power lab system (PowerLab 4/25, ADInstruments). The Tendon Hammer contained a piezo-electric sensor within the head to provide a momentary pulse when a surface is struck with the hammer.

### Analysis

#### Video and ultrasound

We pilot tested the procedures used in other studies (Fletcher et al. 2010; Kay and Blazevich, 2009). During this process, it became clear that we needed to modify our procedures to determine tendon length. The previous studies fixed the ultrasound probe to the belly of the *medial gastrocnemius* to image the attachment of the distal portion of a muscle fascicle into the deep aponeurosis, where changes in tendon length were inferred from displacement of the muscle fascicles, with the assumption that the aponeurosis distal to the measurement site remained a constant length, which we found was not the case. To avoid the fixed length assumption and to image the muscle-tendon junction more accurately, we manually held the ultrasound probe over the MG/AT MTJ and used video to track the location of the ultrasound markers on the probe (Figure 2b), which was digitized using MaxTRAQ software (Figure 2c). This procedure permitted adjustments to the ultrasound coordinates due to any tilt of the probe to be made by multiplying the vertical coordinates of the ultrasound image by the digitized distance between the base of the probe and the midpoint of the top of the probe (Figure 2b) by the actual distance between those points. By doing this we were able to precisely locate the muscle-tendon junction in absolute space while making no assumptions about the length of the aponeurosis. Reliability of thee method was assessed by test-retest of 4 participants across 3 trials with a typical error measurement (within-subject standard deviation) of tendon length of 2.2%.

#### Tendon length and stiffness

Tendon length (TL) was calculated as the distance between the calcaneous marker and the MG/AT MJT in absolute space. Based on the assumption that the effect of the dorsiflexor muscles was minimal, Tendon Force (TF) was estimated by dividing the torque about the ankle joint, obtained from the Biodex with the torque caused by the weight of the foot subtracted, by the moment arm of the Achilles tendon, *d*. The moment arm was calculated using the equation

where

 is a vector containing the position of the markers in absolute space, with *mt* being the musculotendinous junction, *c* being the calcaneus, and *m* being the medial malleolus. Only the portion of each trial where the tendon force was increasing was analyzed. Tendon stiffness was calculated by fitting the following equation to the estimated tendon length and force:

where TF and TTL are the calculated tendon force and tendon length measure respectively, and *F*_0_, *Ae*, and *λ* was fit using the lsqnonlin function in MatLab. The variable λ was used as a measure of the stiffness. The typical error for λ was 16.8%.

**Figure 3:**
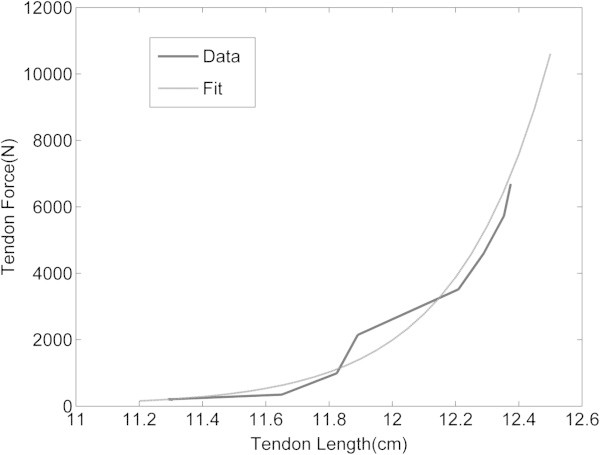
**Example of a typical force-length relationship in the Achilles tendon resulting from a maximal isometric contraction in neutral anatomical position.** The figure illustrates the appropriateness of the exponential curve fit aligned to the raw data.

To improve precision, only contractions with an R^2^ >0.3 (large correlation; 697 of 960 contractions) were used in the tendon stiffness analysis. A lower correlation was deemed too variable for the TF/TL slope. The datasets with R^2^ > 0.5 reduced data points but did not affect the AQUA TITAN tape outcome, but R^2^ > 0.7 resulted in failure of the mixed model procedure to converge due to insufficient data points. Estimates of tendon stiffness previously used linear models (Lichtwark et al. 2007). However, we and others (Lieber et al. 1991; Magid and Law 1985; Pinto and Fung 1973; Winters 1990) observed an exponential length-tension relationship for tendon dynamics during contraction, illustrated in Figure 3. Our exponential fit approach was therefore similar to Hill type muscle models (Winters and Stark 1987).

#### Short latency reflex

The sEMG signal was amplified (BioAmp, ADInstruments, Australia), low band-pass filtered (10–500 Hz) and integrated in Chart for Windows (version7). The onset of reflex sEMG activity was defined as the time between the tendon tap (perturbation) and the first deflection from baseline electrical activity and was determined by visual inspection using a cursor on the display (Grey et al. 2002). The typical error was 4.8%.

### Statistical analysis

The effect of treatment on outcomes was estimated with mixed modelling (Proc Mixed, SAS Version 9.1; SAS Institute, Cary, NC). All data were log-transformed before modelling to reduce nonuniformity of error and to express outcomes and confidence limits (CL) as percentages (Hopkins et al. 2009). Estimates for the effect of treatment on tendon stiffness were derived from the least-squares mean interaction of the model terms (fixed effects) trial order, treatment, post-pre difference, and contraction number (1 and 2); random effects were subject interacted with contraction number and treatment. Estimates for ROM, short latency reflex, and peak isokinetic torque were derived from a model but without contraction or tap number due to trivial difference in the magnitude of the intra-sample means, that is, the value provided by the model was the average of the contractions or tap number. Peak isokinetic torque was estimated using the model approach as for ROM, but for each of the 4 levels of angular torque and overall. Statistical inference was by magnitude-based clinical inference (Hopkins et al. 2009), with the between-subject standardized difference (modified Cohen’s *d*) used as the reference to effect size.

## Results

**Figure 4:**
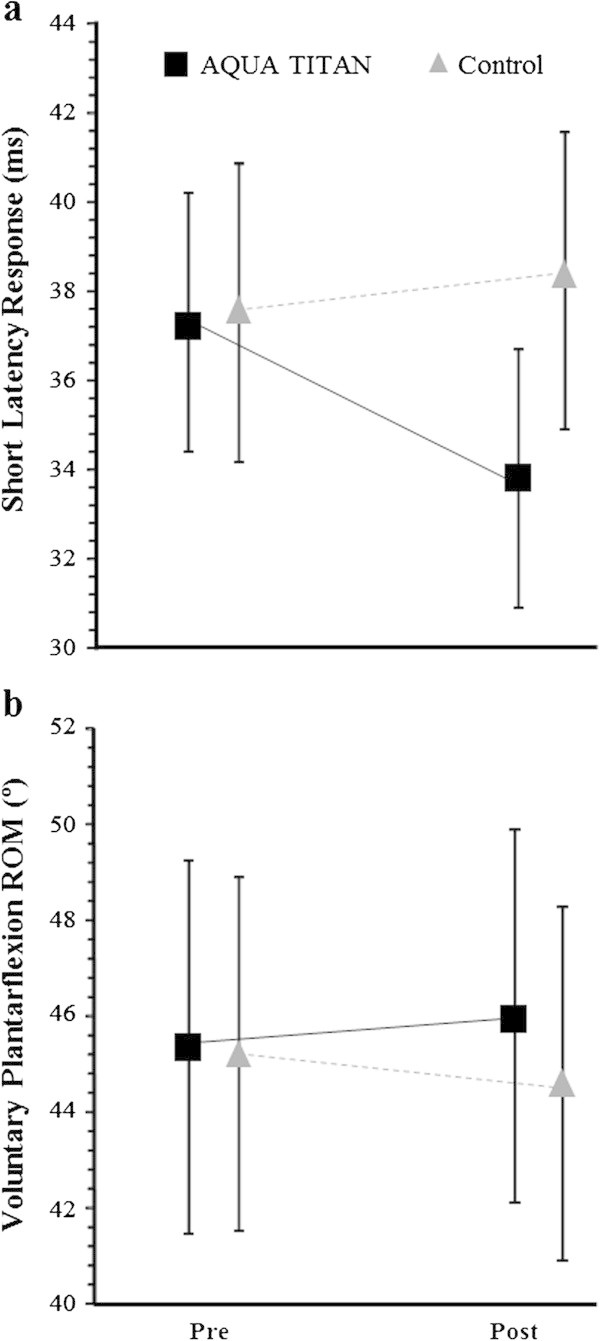
**Effect of 48-h AQUA TITAN tape application following high-intensity intermittent treadmill running on tendon-tap stretch reflex response time and joint range of motion.** Data are **(a)** short latency reflex; **(b)** voluntary plantarflexion ROM. Data are raw means. Variability bars are the between-subject SD.

**Figure 5:**
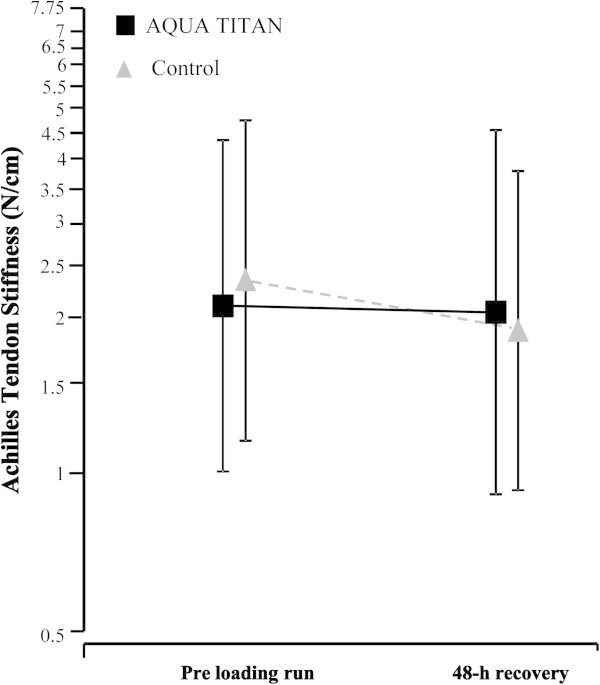
**Effect of AQUA TITAN tape on overall average Achilles tendon stiffness measured during maximal isometric contraction pre and post high-intensity intermittent treadmill run.** Data are the back log-transformed average mean stiffness of all angles measure at maximal force. Variability bars are the between-subject SD.

AQUA TITAN tape led to a small increase in plantarflexor ROM (3.1% 95% CL: ±2.7%) (Figure 4b). A small clear reduction in tendon stiffness (-16.5% ±8.1%) was observed with placebo tape at 48-h post run, but this effect was trivialised with AQUA TITAN (-5.9% ±9.2%) (Figure 5 and Table 1). In contrast, the change in short latency reflex time was negligible with placebo tape (1.6% ±3.8%) when compared to an almost certain large reduction with AQUA TITAN tape (-11.3% ±3.3%) (Figure 4a). The effect of AQUA TITAN tape on peak isokinetic force was trivial (Table 1).

**Table 1:** **Statistical summary of the effect of AQUATITAN tape applied during recovery from intermittent high-intensity running on Achilles tendon stiffness, ankle joint range of motion during plantarflexion, and the short latency reflex, relative to the pre-run baseline, and the post-treatment comparisons for peak torque during maximal isokinetic contractions of the Triceps Surae**

Outcome^1^	Mean effect; ±95%CL(%)^2^	Standardised difference; ±95%CL	P-value	Magnitude-based inference^3^
	Achilles tendon stiffness			
Placebo	-16.5; ±8.1	-0.40; ±0.21	32E-5	Small very likely
AQUA TITAN	-5.9; ±9.2	-0.14; ±0.23	0.23	Trivial possible
AQUA TITAN- Placebo	-11.3; ±11.6	-0.28; ±0.31	0.09	Small possible
	Plantarflexion range of motion			
Placebo	-1.6; ±1.9	-0.12; ±0.15	0.12	Trivial likely
AQUA TITAN	1.6; ±2.0	0.12; ±0.15	0.11	Trivial likely
AQUA TITAN- Placebo	-3.1; ±2.7	-0.24; ±0.21	0.03	Small possible
	Short latency reflex time			
Placebo	1.6; ±3.8	0.20; ±0.48	0.42	Small possible
AQUA TITAN	-11.3; ±3.3	-1.4; ±0.47	8E-9	Large almost certain
AQUA TITAN- Placebo	14.6; ±6.2	1.8; ±0.7	26E-7	Large almost certain
	Peak torque during maximal isokinetic contraction			
30º∙s^-1^ AQUA TITAN- Placebo	4.2; ±14.2	0.06; ±0.19	0.57	Likely trivial
60º∙s^-1^ AQUA TITAN- Placebo	11.1; ±10.9	0.12; ±0.12	0.03	Likely trivial
90º∙s^-1^ AQUA TITAN- Placebo	-7.3; ±8.2	-0.08; ±0.09	0.09	Very likely trivial
120º∙s^-1^ AQUA TITAN- Placebo	-6.2; ±13.8	-0.07; ±0.15	0.41	Likely trivial
Overall AQUA TITAN- Placebo	0.08; ±7.7	0.001; ±0.08	0.98	Almost certainly trivial

^1^Data are the difference in the post intermittent high-intensity run measure minus the pre run measure. For peak torque during maximal isokinetic contraction, only the post-pre difference in the AQUA TITAN minus Placebo contrasts is shown.

^2^Add or subtract this value by the mean to obtain the upper and lower confidence limits.

^3^Magnitude-based inferences about the true value for outcomes were qualified using a modification of the Cohen effect size classification system (trivial = 0.0–0.2, small = 0.2–0.6, moderate = 0.6–1.2, large = 1.2–2.0, very large = 2.0–4.0, and extremely large >4.0). The threshold standardised difference considered substantial is small. The thresholds for assigning qualitative terms to probability of a substantial effect were: <0.5%, almost certainly not; <5%, very unlikely; <25%, unlikely; <75%, possible; >75%, likely; >95%, very likely; >99.5%, almost certain (Hopkins et al. 2009).

## Discussion

In the current study, we utilised an isolated triceps surae model to determine the effect of incorporation of AQUA TITAN titanium micro particles to the flexible tape on lower limb neural-musculotendinous function during recovery from strenuous running. AQUA TITAN-tape application caused a large reduction in short latency reflex time and attenuated the reduction in Achilles tendon stiffness seen with the non-treated control tape 48-h into recovery. These observations suggest that the AQUA TITAN-treated tape leads to faster neuromuscular response to tendon load stimuli that is associated with restoration of tendon stiffness to the pre-loaded condition. The increase in plantarflexion ROM was consistent in magnitude to previous observations (Wadsworth et al. 2010) suggesting the effect of AQUA TITAN on ROM is robust, but small. The effect of AQUA TITAN tape on peak isokinetic force, however, was unaffected leaving the pre-study hypothesis that increased contractility was a mechanism for increased ROM unresolved.

These observations support the hypothesis that AQUA TITAN-treated material applied to the skin better maintains contractile function of the muscle-tendon complex following heavy loading. Stiff tendons are advantageous for performing accurate and repetitive cyclical movements (Alexander 2002; Lichtwark and Wilson 2005) and transmission of muscle shortening to joint movement, whereas a highly compliant tendon may contribute to greater energy return in stretch-shortening contractions (Alexander 2002). For tasks such as running, where the Achilles tendon does not undergo a substantial pre-stretch (Lichtwark et al. 2007), it would be expected that maintenance of tendon stiffness would lead to less decrement in contractile-unit performance with fatigue. Therefore, the new information on reflex control of tendon stiffness may partly explain improved running metabolic efficiency with AQUA TITAN garments (Rowlands et al. 2013; Wadsworth et al. 2010). As a point of reference to effect magnitude, small-moderate (standardized difference: 0.56 to 0.8) increases in tendon stiffness were observed in response to prolonged periods (8–12 weeks) of isometric training (Kubo et al. 2002, 2001). The attenuation in the fatigue-induced stiffness response induced with only 48-h tape intervention, is therefore, noteworthy.

The large reduction in short latency reflex suggests that AQUA TITAN tape has a neurological effect on muscular coordination. Korte (2008) reported that mouse hippocampal pyramidal neurons mounted on a slide under AQUA TITAN tape had lower resting-membrane potential and dose-sensitive firing rates reducing the capacity for long term potentiation induction. Since the short latency reflex is dependent on the balance of excitatory and inhibitory inputs from receptors and in turn modulates the excitability of motor neurons (Hultborn et al. 1987), the present faster tendon-tap reflex suggests that faster nerve conduction through a reflex arc might improve the in vivo peripheral motor control. The peripheral response may, via afferent feedback networks, also influence central motor centres increasing the potential for alterations in postural balance or cyclic tasks that utilize acute feedback to improve contractile function or provide musculoskeletal stability during movement (Ishikawa and Komi 2007).

In addition, reflex latency can modulate muscle-tendon complex stiffness by activation of the spindle during stretch associated with contraction (Cronin et al. 2011; Kay and Blazevich 2009). Cronin et al. (2011) used vibration to impair the triceps surae short latency reflex resulting in greater ankle yield during the support phase of running and reduced force transfer efficiency. Tendon stiffness itself could also affect the contribution of afferent feedback in response to rapid perturbations that elicit short latency reflexes (Cronin et al. 2011). Moreover, feedback from group II afferents in the muscle spindles and Golgi tendon organs were suggested to make an important contribution to running mechanical efficiency (Mazzaro et al. 2005). Together these data suggest that faster short latency reflex with AQUA TITAN may improve gait efficiency independent of tissue adaptation and further investigation into the effect on the peripheral nervous system is warranted and the role this may have in motor tasks and fine neural control of contractile stiffness.

AQUA TITAN-treated material is shown to exert effects on physiology even when not in direct contact with nerves, this is supported by several lines of evidence in cells cultured in plates above pico- to micrometer thickness of titanium: with evidence of enhanced osteoblast differentiation (Sugita et al. 2011), migration, proliferation, and differentiation of myoblasts (Ishizaki et al. 2011) and altered neuronal firing rate (Korte 2008). That the effects of titanium are inhibited when cells are shielded with aluminium wrapping and lead plates in both animal and culture studies (Aoi et al. 2009; Korte 2008), indicates that the influence of AQUA TITAN on neurons seems to be mediated via some factor that can cross open space but does not penetrate other metals, such as electromagnetic waves. This is further supported in a recent in vivo study where altered physiological stress responses were measured following five days sleeping in a room containing titanium (Aoi et al. 2012). Further research is required to determine the magnitude and clinical effects of longer-term exposure to wearable garments treated with AQUA TITAN, while information on dose response and on the physical and physiological mechanisms will be valuable in determining if there is a robust evidential base for application.

Another possible mechanism for the changes observed with AQUA TITAN is improved thermal conductivity. Previously, participants reported increased thermal comfort with AQUA TITAN garments (Wadsworth et al. 2010). Titanium dioxide has a relatively moderate thermal conductivity of 5.8 W·m^-1^·K^-1^ (compared to 0.6 W·m^-1^·K^-1^ for water, 440 W·m^-1^·K^-1^ for silver, and 220 W·m^-1^·K^-1^ for aluminium). Thus, the titanium particles in AQUA TITAN tape may act as an affecter for tissue thermoregulation. Heat plays a major role in the function of physiological processes: in hyperthermia, muscles are able to produce less force (Rall and Woledge 1990), tendon stiffness decreases (Ettema and Huijing 1994), and nerve performance is impaired (De Vrind et al. 1992). By maintaining a more consistent temperature during contraction, altered material thermal conductivity could explain the improvements in motor reflex latency, ROM, and maintenance of tendon stiffness reported here. Therefore, studies on the effect of AQUA TITAN on skeletal muscle and tendon temperature during contractile activity are warranted to examine the thermal conductivity hypothesis.

### Limitations

Our insight, attention to double-blinding, and resource limitations when designing the current experiments was not sufficient to consider including a non-taped condition within the crossover. In the review process, it was brought to our attention that because of the potential for taping to enhance proprioceptive acuity by increased stimulation of the cutaneous mechanoreceptors (Feuerbach et al. 1994), a non-taped condition should be considered in future research. A non-taped condition would permit quantification of the magnitude of the tape effect next to the AQUA TITAN-tape mediated tendon tap response, although masking a placebo effect could be difficult to resolve. A second limitation was our assumption that the dorsiflexor moment was minimal and that the recorded moment was equal to the moment from the dynamometer. Magnusson et al. (2001) found there was minimal change in calculated tendon force based on a correction for dorsiflexor coactivation during maximal plantarflexion. Given that the study sought to compare AQUA TITAN-treated tape against non-treated placebo tape, and that neither tape covered the tibialis anterior, there was a priori no clear reason to suspect greater tibialis anterior activation. Nevertheless, the possibility that AQUA TITAN-tape may affect efferent motor nerve activity via a supraspinal mechanism is worthy of further investigation.

With respect to the tendon moment arm, our calculations were based on the location of skin markers as there was no imaging of the ankle joint during the movement. Absence of imaging removed the possibility of determination of the moments. Other researchers (Fletcher et al. 2010; Kay and Blazevich, 2009) calculated the moment arm as the ratio of tendon shortening to angular displacement of the joint; however, this also requires accurate measurements of the ankle angle, which would have had to be determined by skin markers, and therefore creating similar innate errors to our present method. The ultrasound and recording systems were triggered by the same source, which meant that the starting point for subsequent analysis was in unison. The camera was synchronized using the first frame in which a light appeared giving accuracy to within 1/30 of a second; however, future studies should attempt increased temporal synchronisation.

## Conclusion

The application of AQUA TITAN-treated tape to the triceps surae complex during and following strenuous running decreased short latency reflex response time and maintained Achilles tendon stiffness, relative to untreated placebo tape. The inference from the short latency reflex outcome suggests faster reflex motor control as the most likely mechanism responsible for the tendon response, which might improve musculotendinous performance. These findings may explain, in part, faster restoration of running economy reported with application of AQUA TITAN-treated garments during recovery from strenuous exercise. A greater understanding of the physical mechanism of action of dermally-applied AQUA TITAN-treated materials on the physiological processes influencing musculotendinous recovery is required to provide further evidence in support of the restoration hypothesis.
